# Height to first pod: A review of genetic and breeding approaches to improve combine harvesting in legume crops

**DOI:** 10.3389/fpls.2022.948099

**Published:** 2022-09-16

**Authors:** Marzhan Kuzbakova, Gulmira Khassanova, Irina Oshergina, Evgeniy Ten, Satyvaldy Jatayev, Raushan Yerzhebayeva, Kulpash Bulatova, Sholpan Khalbayeva, Carly Schramm, Peter Anderson, Crystal Sweetman, Colin L. D. Jenkins, Kathleen L. Soole, Yuri Shavrukov

**Affiliations:** ^1^Faculty of Agronomy, S. Seifullin Kazakh Agro Technical University, Nur-Sultan, Kazakhstan; ^2^A.I. Barayev Research and Production Centre of Grain Farming, Shortandy, Kazakhstan; ^3^Kazakh Research Institute of Agriculture and Plant Growing, Almalybak, Kazakhstan; ^4^College of Science and Engineering, Biological Sciences, Flinders University, Adelaide, SA, Australia

**Keywords:** auxin transport and signal transduction genes, *BEBT* or *WD40* genes, candidate genes, gene expression, height to the first pod, MADS box gene *SOC1*, QTL analysis

## Abstract

Height from soil at the base of plant to the first pod (HFP) is an important trait for mechanical harvesting of legume crops. To minimise the loss of pods, the HFP must be higher than that of the blades of most combine harvesters. Here, we review the genetic control, morphology, and variability of HFP in legumes and attempt to unravel the diverse terminology for this trait in the literature. HFP is directly related to node number and internode length but through different mechanisms. The phenotypic diversity and heritability of HFP and their correlations with plant height are very high among studied legumes. Only a few publications describe a QTL analysis where candidate genes for HFP with confirmed gene expression have been mapped. They include major QTLs with eight candidate genes for HFP, which are involved in auxin transport and signal transduction in soybean [*Glycine max* (L.) Merr.] as well as MADS box gene *SOC1* in *Medicago trancatula*, and *BEBT* or *WD40* genes located nearby in the mapped QTL in common bean (*Phaseolus vulgaris* L.). There is no information available about simple and efficient markers associated with HFP, which can be used for marker-assisted selection for this trait in practical breeding, which is still required in the nearest future. To our best knowledge, this is the first review to focus on this significant challenge in legume-based cropping systems.

## Introduction

Grain legumes (or pulses) are very important crops that have been providing essential components of human foods and diets for hundreds of years, including many micronutrients present at only minimal levels in modern varieties of more widely grown cereal crops (FAO, 2016). Higher yield and better quality are the main targets of crop breeding both in favourable and stressed conditions. However, to truly reap the benefits of advances in this area, it is important to minimize yield losses during harvesting. In both cereals and legumes, major challenges at harvest are plant lodging and pod dehiscence, respectively; but in legumes, the position and distribution of pods on the stem is also an important factor, which is botanically very different from spikes in cereals.

Height from the soil at the base of the plant to the first pod, or height to first pod (HFP), is a trait of key importance when using a mechanical combine harvester (Kowalczuk, 1999; Fratini et al., 2007). If the cutterbar level is too low, even with modern harvesters, it can be damaged physically by stones or other debris on the soil surface. However, as was reported in various cultivars of soybean [*Glycine max* (L.) Merr.], if the remaining stubble height is above 15 cm, lower pods will not be collected, resulting in net yield loss (Kang et al., 2017). Martin and Wilcox (1973) estimated a 3–14% loss in seed yield, with a further 4% reduction in soybean yield for every 2.5-cm increase in the cutterbar level. Such loss in soybean seeds can be as dramatic as 7–8-fold with a cutterbar level higher than 15 cm, but an optimised cutting level together with a medium level of sowing density (80 seeds per m^2^), and sufficient distribution of rainfall, provide the best yield (Rêbilas et al., 2020).

In addition to HFP, the distribution of pods along the stem of a legume plant is very important and may even be more important for the total loss of pods with seeds during combine harvesting. This is simply because plants with relatively low HFP will result in minimum seed loss if the rest of the pods are mainly distributed in the medium-upper part of the stem. In contrast, a much stronger impact and seed loss could be recorded in other plants with higher HFP if the majority of pods are grouped in the lower-medium part of the stem (Eckert et al., 2011a,b). A recent model described a perfect relationship between theoretical calculations and experiments in field trials (Rêbilas et al., 2020).

To minimise the yield loss of seed from pods below the cutter level, the HFP must be reasonably high, i.e., at least 12 cm, in soybean (Ramteke et al., 2012). In other crops, i.e., in common bean (*Phaseolus vulgaris* L.), the HFP was indicated as 15 cm for mechanical harvesting (Bisognin et al., 2019), while in chickpea (*Cicer arietinum* L.) the optimum margins for HFP were estimated to be much higher, from 25 to 29 cm, to ensure no seed is lost (Petrova, 2021). In contrast, plants with low HFP are undesirable for combine harvesting of pulse crops but perfectly suitable for vegetable legumes (reviewed in Dhaliwal et al., 2020) in small farms utilising manual harvest techniques or for grazing. It is important to note that widely used legumes like soybean, chickpea, common bean, and lentil (*Lens culinaris* Medik.) remain in the focus of plant breeders and biologists, where HFP is relatively well-studied and reported in publications. However, despite a very intensive search on available databases, no or only rare published information about HFP was found in minor legumes, including pigeon pea [*Cajanus cajan* (L.) Millsp.], mung bean [*Vigna radiata* (L.) R. Wilczek], black gram [*Vigna mungo* (L.) Hepper], and many others. In pulse-growing areas, more and more crops are now being mechanically harvested, where HFP remains a very important trait, but in the absence of published information, the deserved discussion of HFP in minor legumes is very limited.

This critical review shows both the current status and gaps in our knowledge of possible physiological mechanisms and genetic background for HFP in legume plant species. If practical plant breeding for the trait is to be realised in the future, a thorough review of the genetic and physiological control of HFP is needed. Within the current literature on HFP in legumes, we saw major gaps and many conflicting terminologies, methodologies, and ways of assessing this trait. Therefore, the current review is intended as a necessary starting point on the way to a more cohesive and widely relevant view and study of the topic.

## Terminology: Different names with the same meaning

It is important from the beginning to designate a term to describe HFP to avoid any potential misunderstanding. Which term is best used to describe ‘the distance from soil at the base of plant to the first pod (or node with the first pod) produced in legume plants’? This seemingly simple question is not as trivial as it appears. For example, the singular term ‘harvest index’ is only used worldwide while linguistic synonyms are widely accepted for ‘plant height’ (PH) or ‘height of plant.’ However, the terminology for synonyms of ‘height to first pod’ (HFP) or ‘first pod height’ (FPH) is dramatically different. The diversity of terms describing this trait is vast and creates problems in literature searches for very different terms with the same meaning. A list of typical (but not comprehensive) terms used is presented in Table 1, where authors referred either to nodes (height or number) or pods (insertion position or height).

**TABLE 1:** Terms used for the same traits of distance from soil at the base of plant to the first pod, or node with the first pod, produced in legume plants.

Name and abbreviation	Reference	Country of corresponding author
***Node (height)***		
Height at the first node (HFN)	Fratini et al., 2007	Spain
Height of the first reproductive node (H1RN)	Gómez and Ligarreto, 2012	Colombia
Height to first fertile node (HFFN)	Kalapchieva et al., 2020	Bulgaria
Height to the first fruiting node (HFFN)	Gupta et al., 1983	India
Height to first flower node (HFFN)	Hassanpour and Sahhafi, 2020	Iran
Height of lowest pod-bearing node (HLPBN)	Nasto et al., 2016	Albania
Length of first fruiting node (LFFN)	Singh et al., 2019	India
***Node (number)***		
First node of flower and First node of flower initiation (NFI)	Wenden et al., 2009	France
First blossom node (FBN)	Singh and Dhall, 2018	India
First fertile node insertion (FFNI)	Gresta et al., 2016	Italy
Nodes to first flower (NFF)	Xu et al., 2013; Jaudal et al., 2018; Nkhata et al., 2020	China; NZ-United States; South Africa
Node of first flower (NFF)	Catt et al., 2017	Australia
Node number of first flower (NNFF)	Erskine et al., 1994	Syria
Node number subtending the first pod (NNSFP)	Basnet et al., 1972	United States
***Pod (insertion)***		
Height of the first pod insertion (HPI)	Zilio et al., 2013; Dallastra et al., 2014; Soratto et al., 2020	Brazil
First pod insertion height (FPI and FPIH)	Costa et al., 2008; Bisognin et al., 2019; Delfini et al., 2021	Brazil
Insertion height of first pod (IHFP)	Azevedo et al., 2021	Brazil
Insertion height for the first pod (IHFP)	Luiz et al., 2020	Brazil
Intersection height of the first pod (IHFP)	Teixeira et al., 2017	Brazil
Insertion height of the lowest pod (IHLP)	Ramteke et al., 2012	India
***Pod (height)***		
First pod height (FPH)	Robertson et al., 1997; Yilmaz, 2003; Özveren et al., 2006; Öz, 2008; Togay et al., 2008; Basaran et al., 2011; Kosev and Mikiæ, 2012; Kang et al., 2017; Jiang et al., 2018; Tabti et al., 2018; Kim et al., 2019; Celik and Boydak, 2020; Nadeem et al., 2020; Patinni et al., 2020; Ahmad et al., 2021; Girgel, 2021; Güzel and Özyazıcı, 2021; Kosev and Georgieva, 2021; Uçar et al., 2021; Santana et al., 2022	Argentina; Brazil; Bulgaria; China; Iraq; Korea; Syria; Turkey
Height to the first pod (HFP)	Petrova, 2021; Staniak et al., 2021	Bulgaria; Poland
Height of first pod (HFP)	Epler and Staggenborg, 2008; Amri-Tiliouine et al., 2018; Tabti et al., 2018; Sobko et al., 2019, 2020; Rêbilas et al., 2020	Algeria; Argentina; Germany; Poland; United States
Height of the first pod setting (HFPS)	Rêbilas et al., 2020	Poland
Height of the bottom pod (HBP)	Ramteke et al., 2012; Wu et al., 2015	China; India
Height of the lowest pod (HLP)	Gan et al., 2003a,b; Akash et al., 2017; Qin et al., 2017	Canada; China; Jordan
Height of the lowest pod setting (HLPS)	Kowalczuk, 1999	Poland
Lower pod setting height (LPSH)	Seferova and Bulakh, 2019	Russia
Lowest pod height (LPH)	Martin and Wilcox, 1973; Grabau et al., 1991; Acquaah et al., 1992; Öz, 2008; Eckert et al., 2011a,b; Kato et al., 2018, 2019	Japan; Turkey; United States
Basal pod height (BPH)	Biçer and Şakar, 2008; Singh and Vatsa, 2009; Karami, 2011; Khadka et al., 2021	India; Iran; Nepal; Turkey
First pod setting height (FPSH)	Borowska and Prusiñski, 2021	Poland
Distance to the first pod (DFP)	Miladinović et al., 2021	Serbia

As can be summarised from the top-part of Table 1, there is no or very little consensus regarding the use of ‘nodes’ in reference to the trait, because unique and ‘personalised’ terms are used in each case. Only a few exceptions were found with ‘node number,’ but this is not the same as ‘height to node,’ as will be discussed later.

In contrast, as shown in the bottom part of Table 1, many reports in the literature use the same or similar terms for ‘pods,’ where FPH (first pod height) is clearly the most commonly used, while the terms for ‘bottom,’ ‘lowest,’ and ‘basal’ pods are synonymous. Interestingly, terms referring to ‘pod insertion’ were used by almost all researchers from Brazil (with one exception), and it is likely a reflection of a translation of the terms from Portuguese to English. Other groups of terms have no geographic association (Table 1).

For the purpose of the present review, only the term height to first pod (HFP) will be used as the synonym for ‘distance from soil level to the first pod (or node with first pod) produced in grain legume plants.’ However, no articles were excluded from our analysis based on differences in the terminology.

## Internode length and node number

During plant development, flower primordia are initiated at certain nodes. This is an important and complex biological process that is tightly regulated to produce the best reproductive strategy. For example, flowers cannot set and produce pods in the initial (lower) nodes on a stem simply because a young plant has insufficient accumulated nutrients to support pollinated flowers and developing seeds. Therefore, the reproductive period of the plant is optimised for a node number at which the first flower will develop pods with seeds. The physiological response of plants to photoperiod and changes in plant hormone balance can determine and alter the first node of flowering (Tucker, 2003; Hofer and Noel-Ellis, 2014; Benlloch et al., 2015).

In grain legumes, smallest node numbers with first flowers and developing pods varied not only in different genotypes but also in response to a changing environment. For example, in faba bean (*Vicia faba* L.), nodes 6.3–12 and 22.8–28.7 were developed until the first flower in long day and short day conditions, respectively (Catt et al., 2017). Similar significant differences in nodes to first flower were reported across 60 accessions of common bean in response to day-length tested over 2 years and ranging from nodes 5.3–9.7 (Nkhata et al., 2020). However, in other experiments with common bean, temperature was shown to be a main factor determining the node addition rate or how quickly new nodes appear on the stem during plant growth (Zhang L. et al., 2017). Two sowing dates resulted in a smaller difference of nodes 8.6–9.7 with the first flower in a lentil germplasm collection (Erskine et al., 1994).

**FIGURE 1:**
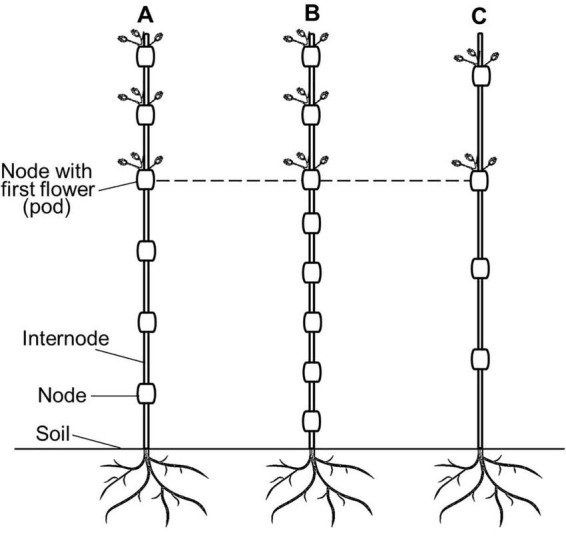
Schematic presentation of nodes with first flowers (pods) occurring in plants with different node numbers and internode lengths. **(A)** Regular nodes and internodes. **(B)** Greater number of nodes with shorter internodes. **(C)** Fewer number of nodes and longer internodes. Dashed line indicates the same height from soil to the nodes with flowers (pods).

Node number to the first flower (or pod) is not the only factor determining HFP but, together with internode length, can provide a complete description of HFP (Figure 1). Of course, both node number and internode length impact on HFP, while different mechanisms are involved in their establishment. Node number is the occurrence of new morphological structures, while internode length is related to cell elongation and division processes. Internode length is determined by elongation (reviewed in McKim, 2020), and how internodes change during plant development was explored by computer analysis of forage legume plant species *Stylosanthes scabra*. The authors reported on the observed non-linear variation of internode length with node order at maturity stage (reduced at nodes 1–3 and 10–20 and increased at nodes 3–10 and 20–30), which was matched with the computer prediction (Wilson et al., 1999).

In general, many traits are involved in plant architecture in legumes. Regarding FPH, three factors were identified based on common bean analysis as follows: (1) elongation factor with internode length, (2) sturdiness factor with hypocotyl length (‘from soil level to base of first branch’), and (3) reproductive factor with ‘location of nodes on which pods were borne’ (Acquaah et al., 1992; Kelly, 2001). Internode length factor is directly involved in FPH formation, while hypocotyl length can partly interact since HFP and to first branch overlap but are not the same. The interaction of a third factor about node location including FPH was also significant. All three factors are variable but showed positive relationships with FPH in response to recurrent selection in common bean, from original parental genotypes to advanced breeding lines (Acquaah et al., 1992).

## Height to first pod: Phenotypic variability, heritability, and correlation with plant height in legumes

**FIGURE 2:**
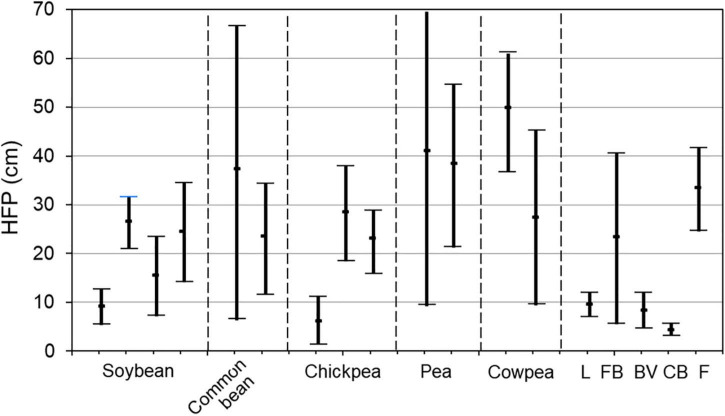
Summary of variability of HFP in legumes from crops indicated on the *x*-axis. As multiple data sets were available for four species (i.e., soybean, common bean, chickpea, and pea), these were separated into segments by dashed lines. The fifth segment contains species for which only two or a single data set was available. L, lentil; FB, faba bean; BV, bitter vetch; CB, cluster bean (guar); F, fenugreek. Each bar represents the minimal, maximal, and average values of HFP from separate articles. Complete detailed data are presented in Supplementary Table S1.

Height to first pod can vary significantly both within and between legume plant species (Figure 2). However, the variability in HFP reported in the literature is strongly dependent on the reproductive biology of the plant species (self- or cross-pollinated), experiment design, calculation method, population size or number of accessions included in the study, and their background, i.e., cultivars, breeding lines, or germplasms (Supplementary Table S1). Therefore, the presented results on heritability and correlations from all published articles are aimed to show an overview of the current situation in this topic. Nevertheless, some interesting patterns emerge when comparing different grain legume species. For example, there is a very similar trend among selected soybean cultivars and chickpea accessions with a very low (Robertson et al., 1997; Epler and Staggenborg, 2008), very high (Martin and Wilcox, 1973; Petrova, 2021), or widely distributed range of HFP (Yilmaz, 2003; Amri-Tiliouine et al., 2018). In contrast, extremely wide ranges of HFP were found in a wild germplasm collection of common bean and among selected pea cultivars (*Pisum sativum* L.) (Gupta et al., 1983; Nadeem et al., 2020). Therefore, the HFP among these crops showed some diversity. Lentil, bitter vetch [*Vicia ervilia* (L.) Willd.] and cluster bean (or guar) [*Cyamopsis tetragonoloba* (L.) Taub.] showed very low HFP ranges among wild germplasm collections, while it was very high in accessions of cowpea [*Vigna unguiculata* (L.) Walp.] and fenugreek (*Trigonella foenum-graceum* L.) and very wide among local faba bean genotypes (Figure 2 and Supplementary Table S1).

Heritability of HFP, in general terms, means how variability in phenotypes among plants in a population is determined by genetic background and environments (Nyquist, 1991; Schmidt et al., 2019). In other words, higher heritability indicates a stronger role of genes in HFP variability, while lower heritability means that the proportion of variability in the trait is less controlled by genetic factors. In grain legumes, HFP is quite variable (summary in Table 2 and more details in Supplementary Table S1), but the results can be loosely classified into three groups of heritability as follows: (1) low-moderate, e.g., 0.28–0.55 (lentil); (2) low-high, e.g., 0–0.74 (soybean) and 0.31–0.83 (chickpea); (3) moderate-high, e.g., 0.68–0.92 (common bean), 0.68–0.93 (pea), and 0.97 (faba bean). It appears unclear why, i.e., the heritability of HFP is lower in soybean and lentil genotypes but higher in common bean, pea, and faba beans, and this requires further research. However, one of the reasons might be the various levels of homo- and heterozygosity in different wild accessions, landraces, breeding lines, and cultivars of legumes studied. In general, the majority of germplasm of legume crops are self-pollinated, therefore generating a predictable level of homozygosity, but cross-pollinations are also employed during various breeding methods. Therefore, HFP variability can also be related to the level of self- and cross-pollinations among the genotypes of legume crops (Kumar et al., 2020).

**TABLE 2:** Summary (minimum-maximum) of heritability range (*H*^2^ or *h*^2^) for height to first pot (HFP), correlation with plant height (PH), and recommended HFP for combine harvesting in legume plant species.

Recommended cut height (cm)	*H*^2^ or *h*^2^ (Broad and narrow sense)	*r* (HFP/PH)	Reference
**Soybean [*Glycine max*(L.) Merr]**			
>10–12–15	*H*^2^ = 0.00–0.74	*r* = 0.23–0.71	Martin and Wilcox, 1973; Costa et al., 2008; Ramteke et al., 2012; Kang et al., 2017; Teixeira et al., 2017; Beiküfner et al., 2019; Seferova and Bulakh, 2019; Rêbilas et al., 2020; Sobko et al., 2020; Borowska and Prusiñski, 2021; Khadka et al., 2021
**Common bean (*Phaseolus vulgaris* L.)**			
15	*h*^2^ = 0.68–0.92	*r* = 0.43–0.69	Bisognin et al., 2019; Nadeem et al., 2020; Delfini et al., 2021; Girgel, 2021
**Chickpea (*Cicer arietinum* L.)**			
25–29	*H*^2^ = 0.31–0.83	*r* = 0.24	Özveren et al., 2006; Biçer and Şakar, 2008; Karami, 2011; Amri-Tiliouine et al., 2018; Kushwah et al., 2020; Petrova, 2021
**Lentil (*Lens culinaris* Medik.)**			
	*h*^2^ = 0.28; *H*^2^ = 0.55	*r* = 0.60	Tabti et al., 2018; Ahmad et al., 2021
**Pea (*Pisum sativum* L.)**			
	*h*^2^ = 0.68; *H*^2^ = 0.93	*r* = 0.83–0.89	Togay et al., 2008; Gómez and Ligarreto, 2012; Kosev and Mikiæ, 2012; Singh et al., 2019
**Faba bean (*Vicea faba* L.)**			
	*H^2^* = 0.97	*r* = 0.84–0.86	Bora et al., 1998

The correlation between HFP and PH was low to moderate in chickpea and soybean (*r* = 0.23–0.71) and moderate in common bean (0.43–0.69) and lentil (0.6), but it showed a much stronger correlation (*r* = 0.83–0.89) in pea and faba bean (summary in Table 2 and more details in Supplementary Table S1). This observation of the correlation between HFP and PH in legumes is very important because plants with longer PH can be used as a ‘proxy’ for bigger HFP, particularly in the case of their moderate to strong correlation. This means that taller plants are more or less likely to have higher position of first pod on the stem depending on PH-HFP correlation level. Such a proxy and the relationship between PH and HFP are influenced by the application of nutrients and chemicals, i.e., fertilisers, which is discussed in the following section. This pointed toward common components regulating both HFP and PH on a similar scale and with a stronger correlation. However, there are other cases where HFP was changed regardless of PH or because of their weaker correlation levels, and they indicate a different mechanism of hormonal balance related to internode length and number. Such ‘non-proxy’ situations with low correlations between PH and HFP will also be discussed later in the review.

## Effect of environment, management, and treatment on height to first pod

As indicated above, internode length and node number to first flower can be affected by environment, particularly day length and sowing date. These and other environmental factors are also impacting HFP. For example, in Korean soybean, early sowing date was associated with higher HFP, and late-sown seeds of soybean cv. Seonpung showed a smaller HFP. However, it was also genotype-dependent, and other studied soybean cultivars did not show such an association between sowing date and HFP. Additionally, the HFP of some genotypes strongly interacted with planting distance, PH, and number of nodes as indicated above (Kang et al., 2017).

Describing ‘harvestability’, some researchers include HFP as important component among other traits of grain legume crops making it suitable for combine harvesting (Gan et al., 2003a,b). Plant density is also included in the components of harvestability, and it was reported to strongly affect HFP. In soybean, over a range of lower densities, from 7 to 29 plants per m^2^, HFP raised by 4.1 cm (from 10 to 14.1 cm), respectively, in one cultivar in Turkey (Öz, 2008), and from 10 to 35 plants per m^2^, HFP was increased either from 6.4 to 10 cm or from 10 to 12.5 cm, respectively, in two soybean cultivars studied in the United States (Epler and Staggenborg, 2008). Similar results were reported for cowpea and chickpea, where a density increase from 4.5 to 22.5 and from 20 to 50 plants per m^2^ was accompanied by higher HFP, from 10 to 45 cm, in one cultivar of cowpea grown in Brazil (Soratto et al., 2020), from 19.9 to 21.9 cm and from 22 to 27.1 cm in Desi and Kabuli ecotypes of chickpea studied in Canada (Gan et al., 2003a,b), respectively. At higher densities, from 30 to 90 plants per m^2^, HFP was increased from 9.4 to 13.4 cm on average in four soybean cultivars in Germany (Sobko et al., 2019). In other experiments with row spacing in four environments in the United States, the positions of lowest pods in plants of three pinto cultivars of common bean grown in narrow rows (increased plant density) was significantly higher (2.2 cm) than in intermediate or wide row spacing (1.54–1.86 cm) (Eckert et al., 2011b). However, the distance differences between plants within rows showed a more complicated and non-linear effect for HFP in both soybean (Yilmaz, 2003) and common bean (Bisognin et al., 2019). Seed size was reported to significantly influence HFP and improve harvestability. In the example of chickpea cv. Sanford (Kabuli ecotype), it was shown that plants grown from larger sized seeds (>9 mm in diameter) had 10–13 mm (5%) increased HFP compared to plants grown from smaller seeds (<9 mm in diameter) under field conditions in Canada (Gan et al., 2003b).

Use of conventional or organic cropping systems also had a significant effect on both HFP and PH in six soybean cultivars grown over 3 years in Germany (Beiküfner et al., 2019). Under conventional cropping practice with the standard application of fertilisers and herbicides, the HFP was 10.4 cm compared to the 7.3 cm HFP of plants grown under organic cultivation. Conventional agriculture provided lower seed loss at harvest (25.6%) when compared to the 39.2% seed loss recorded in organic field trials in the same six soybean cultivars. In both cases, however, this is an enormous loss of seeds, and clearly this trait needs to be improved in order to produce higher yield by mechanical harvest (Beiküfner et al., 2019). Higher dosage of nitrogen fertiliser (90 kg per ha) compared to control increased HFP, not strongly but still significant, by 2.7 cm compared to the control in one soybean cultivar studied in Turkey (Öz, 2008).

However, not all fertilisers showed similar effects on HFP in legumes. The application of a Zn-based fertiliser was expected to be important for yield production in two Turkish breeding lines of faba bean, but inconsistent results were obtained with a dosage range of 2.5–7.5 kg/ha (compared to controls) for HFP (Ulukan et al., 2002). All the results presented in this study showed inconsistent patterns depending on year, genotype, and level of Zn application; therefore, a conclusion on the effect of Zn on HFP cannot be made. Nevertheless, this demonstrates an environmental influence on HFP.

The organic fertiliser leonardite is an oxidised form of lignite obtained from coal mines and an important component for sustainable agriculture in Turkey. It has soil amendment potential and positive effects on crop growth and yield (Uçar et al., 2021). Leonardite was applied to soil at a dosage of 1 tonne/ha before sowing seeds of broad bean cv. Salkim. The treatment resulted in increased HFP from 10.1 to 12.7 (cm) and from 11 to 13.2 (cm) (26 and 29%, respectively) over 2 years of tests. Similarly, the PH of broad beans was also increased in a field trial after leonardite application (Uçar et al., 2021). However, applications of fertilisers can have a general response to plant growth and not related to a specific change in HFP; therefore, published results for fertilisers need to be considered more carefully.

Humic acids (HAs) are organic substances of humus extracted from soil and represent a very important component of soil organic matter. HA and humus support soil fertility through the water-holding capacity of the soil, particularly in dry environments (Ulukan et al., 2012a,b). HA was reported to affect equally both the PH and HFP of chickpea cultivars in field trials under semi-arid conditions. However, low and high dosages of HAs applied prior to seed sowing showed exactly opposite effects: stimulating or reducing PH and HFP in chickpea plants (Ulukan et al., 2012a,b).

Ethephon [2-(chloroethyl) phosphonic acid] is a plant growth regulator that is converted to ethylene after plant treatment. Lodging resistance is improved in crops after ethephon application mainly by reducing PH. In soybean, ethephon reduced PH without reducing HFP. The application of ethephon in different stages of ontogenesis reduced PH to a similar degree but did not affect HFP; therefore, it had no effect on harvest loss (Grabau et al., 1991).

In other trials involving soil acidity and cold treatment of soybean, the HFP was not significantly different (Patinni et al., 2020; Staniak et al., 2021). However, in an experiment with *Bradyrhizobium*, by inoculation of six soybean cultivars in Serbia, it was shown that FPH and PH were significantly shorter after inoculation but not in all studied genotypes (Miladinović et al., 2021). In more general terms, abiotic stresses like drought and heat, as well as disease infections, significantly affect plant growth and reduce PH, which was shown in various legumes (reviewed in Parihar et al., 2020; Pratap et al., 2021). As a part of PH, HFP is also reduced because of shorter internode length in plants affected by abiotic and biotic stresses. For example, it was reported that HFP was higher in cowpea plants grown under higher rainfall compared to dry seasons (Basaran et al., 2011). In contrast, sowing seeds in winter can cause over-watering, and in such conditions, HFP and PH were reported to be significantly reduced in lentil and fenugreek plants compared to more favourable seasons (Ahmad et al., 2021; Güzel and Özyazıcı, 2021). We can hypothesise that earlier events with stresses in plant growth can have a stronger reduction of HFP, whereas the later occurrence of stresses can have a lesser impact, since HFP is a trait of the bottom part of the stem which develops earlier. This issue can be clarified after further studies.

Collectively, these data provide some evidence that HFP is highly affected by the environment, which has direct implications for breeding.

### Association with type of stem growth: Erect-prostrate and indeterminate-determinate

Different types of stem growth in plant species, including legumes, can have an indirect influence on HPF, and two such systems are described here. The first system, the ‘erect-prostrate’ geometric positions of plant stems, relates to the biological traits of some genotypes or entire species of legumes. Plant architecture with erect stems is much preferred for combine harvester use, such as most soybean cultivars and some chickpea and bean genotypes that satisfy the requirements for machine harvesting (Kushwah et al., 2020). Additionally, plants with upright stems are shown to be less susceptible to infections of white mold fungi [*Sclerotinia sclerotiorum* (Lib.) de Bary] in common bean due to better aeration between plants and decreased humidity (Soltani et al., 2016). In contrast, prostrate- and semi-prostrate-type legumes, such as cowpea, could be preferable for forage and green biomass production for livestock only (Ravelombola et al., 2017). However, the majority of legumes have a semi-prostrate habit, where both HFP and trigonometric prediction of stem position with pods at harvest have to be taken into consideration (Ramteke et al., 2012). It is important to note that the erect-prostrate type of stem growth has independent genetic mechanisms (Upadhyaya et al., 2017; Zhang B. et al., 2017; Huang et al., 2020). However, recent results revealed that two auxin-related genes (transporter and transport inhibitor) were more active in plants with a prostrate habit than those with erect stems in plants of the perennial legume *Astragalus adsurgens* Pall., a Chinese native forage crop (Ma et al., 2020). The involvement of auxin-related genes will be discussed later in the review.

The second architectural feature is determinate or indeterminate stem growth, and this is mainly controlled by the *Dt1* (Determinate1) gene in different plant species including legumes (Huyghe, 1998). The *Dt1* genes have homology with *TFL1* (Terminal flower 1) in *Arabidopsis thaliana* which changes meristem indeterminacy in shoots, and these genes were widely analysed among tribe Phaseoleae (Krylova et al., 2021). For example, in common bean, the *PvTFL1y* gene was reported as a functional homolog of *TFL1*, and insertion of the transgene *PvTFL1y* in the *tfl1-1* mutant recovered the wild-type growth habit in *A. thaliana* (Repinski et al., 2012). Additionally, unique haplotypes were identified and confirmed in a segregating population for *PvTFL1y* with either retrotransposon or splice-site mutation (Repinski et al., 2012), where a determinate type of growth was consistent with the earlier described recessive *fin* mutant in common bean (Kelly, 2001). Similar *TFL1* homologs were found and reported in genotypes of hyacinth bean or Indian bean [*Lablab purpureus* (L.) Sweet] (Kaldate et al., 2021) and among EMS-induced mutants of mung bean using a TILLING by sequencing approach (Varadaraju et al., 2021). In soybean, *Dt1* orthologues was identified as *GmTFL1* (Glyma19g37890), located on chromosome 19 with two dominant and four recessive alleles. It was reported that HFP was significantly greater in plants with indeterminate and semi-determinate growth compared to those in determinate genotypes (Hartung et al., 1981; Kato et al., 2018). In this case, plants with different type of growth and optimized HFP are required for cultivation of legumes in areas with ridges. Therefore, the change from determinate to semi-determinate growth habit has an advantage in terms of lowest pod height (Kato et al., 2019). A second gene (*Dt2*) significantly reduced plant height, node number, and HFP. Therefore, more pods will be below the combine cutter at harvest and more seeds are lost in soybean genotypes with a determinate growth type (Hartung et al., 1981).

### Plant hormones, QTLs, and genes associated with internode elongation

#### Gibberellic acid biosynthesis, and signalling genes

Over almost a century since the discovery of gibberellic acid (GA), it has become very clear that this plant hormone has an enormous effect on cell elongation in different organs, including the stem, resulting in internode elongation and taller PH (reviewed in Huyghe, 1998; Sun, 2010; Hedden and Sponsel, 2015; McKim, 2020). A massive number of mutants of different genes resulting in GA deficiency, and the controlling biosynthesis pathway of GA, have been described, i.e., 15 different genes in pea (Gupta et al., 1983; Huyghe, 1998). Defects in genes in any stage of GA-related biosynthesis or transduction, or receptors caused dwarf and slender phenotypes of PH with shorter internodes and smaller HFP (Huyghe, 1998; Gómez and Ligarreto, 2012). The coefficient of genetic variation in pea GA mutants widely ranged from 29.3 to 91.2% for HFP (Kalapchieva et al., 2020).

In pea, the *gi* (Gigas) mutant causes non-flowering in plants, with a stable compact stage and strongly reduced internode length. The plant height and internode length were restored after GA treatment (Beveridge et al., 2001). Similarly, pea mutations blocked GA biosynthesis causing a marked reduction in internode length (Ingram and Reid, 1987). The magnitude of the photoperiod response, in terms of number of nodes established prior to flower development, was much the same in GA-deficient plants as in their normal (wild type) counterparts (Murfet and Reid, 1987; Weller and Reid, 1993).

In sweet pea, *Lathyrus odoratus* L., the effect of the semi-dwarf gene *L/l* was obvious and the shorter internodes resulted from a reduction in both the length and number of epidermal cells per internode. In plants with *ll* dwarf genotypes, the node of flower initiation was reduced slightly. The effect of another *lb* dwarf allele was confined largely to internode length (Ross et al., 1990). Similarly, in *Pisum sativum* L., the dominant mutant allele *bsh* has very large effects, and the reduction in internode length was around 10-fold (Symons et al., 1999).

An EMS-induced dwarf mutant (*dw*) in soybean revealed a novel nuclear gene, *GmDW1* encoding *ent*-kaurene synthase, mapped to chromosome 8. This resulted in shorter PH and internodes, but this phenotype was reversed after GA_3_ treatment (Li et al., 2018). A recent report demonstrated that overexpression of *GmGAMYB*, an R2R3-MYB transcription factor, promoted plant height and elongated internodes in soybean plants by positive regulation of the GA-biosynthesis gene *GmGA20ox*, and scanning microscopy confirmed significantly longer internode epidermal cells (Yang et al., 2021).

Internode length was studied on 84 recombinant inbred lines (RILs) from a cross in common bean, and six QTLs were identified for internodes ‘halfway up’ the climbing plant height. Five quantitative trait loci (QTL) overlapped with QTL for PH, with the simple meaning that taller plants have longer internodes but the authors did not study HFP (Checa and Blair, 2008).

Plant height and internode length are also under the control of other genes involved in GA signal transduction (Sun, 2010; McKim, 2020). Additionally, GA interacts with other plant hormones, particularly auxins, resulting in taller PH and longer internodes, as was shown in pea (Ross et al., 2003). In summary, in all cases with GA-related genes, longer internodes and bigger HFP are strongly associated with taller PH, and, correspondingly, semi-dwarf and dwarf mutants always have shorter internodes and reduced HFP.

### Auxin transport and signal transduction genes

**FIGURE 3:**
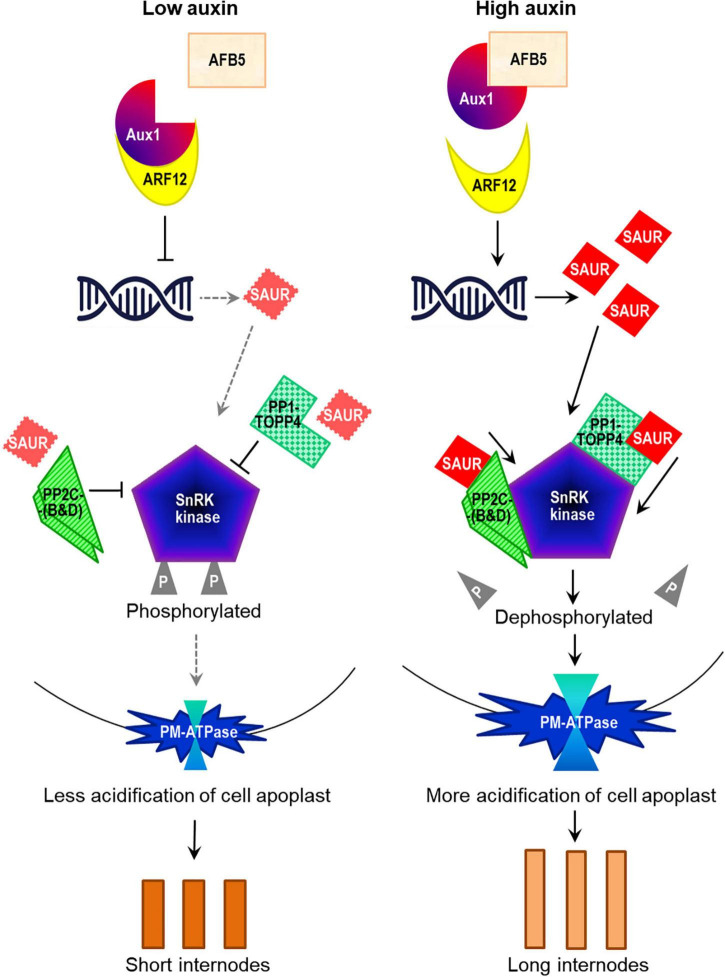
Schematic presentation of polypeptides encoded by eight candidate genes for HFP in soybean involved in auxin transport and the signal transduction genetic network and resulting in internode elongation (based on Jiang et al., 2018). Names of the proteins are based on homologues in *Arabidopsis* as follows (in the order of occurrence): (1) AFB5, auxin F-box-like protein; (2) Aux1, auxin influx transporter-related protein 1; (3) ARF12, auxin response factor 12-related; (4) SAUR, small auxin-upregulated RNA; (5 and 6) PP2C, protein phosphatase 2C-like protein, clades B and D; (7) PP1, serine/threonine-protein phosphatase 1, homologue TOPP4; (8) SnRK, sucrose non-fermenting-related protein kinase. PM ATPase, plasma membrane ATPase, protein pump in cytoplasm. Dashed grey and black solid arrows show weak and strong productions of proteins, respectively. The Figure was generated based on information and Figures from Hou et al. (2016), Taylor-Teeples et al. (2016), Stortenbeker and Bemer (2019), and Du et al. (2020).

From the comprehensive analysis of 147 RILs of soybean originating from a single cross between cultivars Charleston and Dongnong-594, and a molecular map based on 5,308 specific-length amplified fragment (SLAF) markers, Jiang et al. (2018) identified 11 major QTLs with eight candidate genes involved in the control of HFP in soybean (Table 3, Figure 3).

**TABLE 3:** Summary of QTLs and potential candidate gene for HFP in legumes.

QTL	Potential candidate gene	Reference
**Soybean [*Glycine max*(L.) Merr]**		
11 major QTLs, 147 RILs from the cross Charleston × Dongnong-594.	8 candidate genes. (1) *Glyma.07g147000* = *GmAux1-like*, Auxin influx transporter protein 1; (2) *Glyma.02g211800* = *GmAFB5-like*, Auxin F-Box protein; (3) *Glyma.07g134800* = *GmARF12*, Auxin response factor 12; (4) *Glyma.16g129600* = *GmSAUR*, Small auxin-upregulated RNA; (5) *Glyma.02g228200* = *GmPP2C*-like, Protein phosphatase 2C-like, clade B; (6) *Glyma.16G122200* = *GmPP2C*-like, Protein phosphatase 2C-like, clade D; (7) *Glyma.20G222500* = *GmPP1*-like, Serine/threonine-protein phosphatase PP1 isozyme 2; (8) *Glyma.17g178800* = *GmSnRK2*-*like*, Serine/threonine related protein kinase.	Jiang et al., 2018
***Medicago truncatula*Gaertn.**		
Mutant *Mtsoc1a*, short primary stems with shorter nodes to the first flower. Overexpression of *MtSOC1a* caused increase in primary stem height and HFP.	*Medtr7g075870* = *MtSOC1a*, Suppressor of Overexpression of Constants 1, Transcription factor MADS-box family 22.	Jaudal et al., 2018
**Common bean (*Phaseolus vulgaris* L.)**		
Genome-wide association study (GWAS) analysis in a Brazilian diversity panel of 178 accessions.	*Phvul.006G098300* = *BEBT*, Benzyl alcohol O-benzoyltransferase.	Delfini et al., 2021
	*Phvul.006G098200* = *WD40* or *WDR*, WD40-repeat gene. Alternative candidate gene.	Own analysis
**Adzuki bean [*Vigna angularis* (Willd.) Ohwi & H. Ohashi]**		
QTL for stem internode length, chromosome 9. F_2_ mapping population from the cross of adzuki bean cv. Ass001 and a wild accession CWA108.		Li et al., 2017
**Lentil (*Lens culinaris* Medik.)**		
Two QTLs, chromosomes 1 and 5. F_2_ population from the cross of cv. Lupa and accession BG 16880.		Fratini et al., 2007
**Asparagus bean (*Vigna unguiculata* ssp. *sesquipedalis*)**		
Two QTLs for node number to first flower, chromosomes LG11 and LG4. 209 RILs F_8:9_ produced by single seed descent from the cross of cvs. ZN016 and ZJ282.		Xu et al., 2013

The first identified gene is *Glyma.07g147000*, *GmAux1*-like, which encodes auxin influx transporter protein 1. This gene is an orthologue of *Aux1* in *Arabidopsis thaliana* (L.) Heynh., At2g38120 (Taylor-Teeples et al., 2016), which acts as a transcriptional co-repressor with another identified gene, *Glyma.02g211800*, a *GmAFB5*-like encoded auxin F-Box protein (reviewed in: Ho et al., 2008; Williams et al., 2019). Both *Aux1* and *AFB5* genes participate in the nuclear auxin signalling pathway as auxin receptors and regulators (Prigge et al., 2016), and they both act together in co-repressing a third identified gene, *Glyma.07g134800*, *GmARF12*, auxin response factor 12 (reviewed in: Luo et al., 2018; Du et al., 2020). Another identified candidate gene, *Glyma.16g129600* (= *GmSAUR*), a small auxin-upregulated RNA and ortholog of *SAUR-10* in *Arabidopsis*, At4g34760 (reviewed in: Stortenbeker and Bemer, 2019; Du et al., 2020), and in various plant species, including legumes (Li et al., 1994). SAUR proteins have also been shown to inhibit a group of protein phosphatases located in the plasma membrane and, therefore, this group of genes is very important for cell growth regulation (reviewed in: van Mourik et al., 2017; Luo et al., 2018).

There are many different types of protein phosphatases in plants with a range of functions (reviewed in Farkas et al., 2007), but at least two of them are relevant to HFP because they represent three other identified candidate genes. *Glyma.02g228200* and *Glyma.16G122200* are both *GmPP2C*-like, a protein phosphatase 2C-like protein, and orthologues of the *ArabidopsisPP2C*-superfamily clades B and D, respectively (At2g30020 and At3g51370). The third gene, *Glyma.20G222500*, is from another class, *GmPP1*-like, a serine/threonine-protein phosphatase PP1 isozyme 2 and orthologue of *Arabidopsis TOPP4*, type one serine/threonine protein phosphatase 4 (At2g39840) (Zhang et al., 2020). The *topp4-*dwarf mutant has shorter PH and internode length but overexpressed lines with TOPP4 re-introduced into the mutant background showed a reverse effect and recovered plant height and internode length (Qin et al., 2017). For our analysis, it is important to note that the phosphatase activity of all the three *PP2C* and *PPI* genes is inhibited by SAUR proteins.

The last identified candidate gene was *Glyma.17g178800*, a serine/threonine-related protein kinase, *GmSnRK2*-like, and orthologue of *Arabidopsis SnRK2-3*, which is sucrose non-fermenting related protein kinase 2-3 (At5g66880). The SnRK2 enzyme is involved in ABA signalling (Hou et al., 2016; Endo et al., 2018; Lu et al., 2020) and in the regulation of plasma membrane PM H^+^-ATPase by phosphorylation, acidification of cell apoplast and signal transduction for cell elongation (Akiyama et al., 2022), and finally for longer internodes in plant growth (Hager, 2003; Spartz et al., 2014). It was concluded that the results will aid in building a foundation for marker-assisted selection for HFP breeding in soybean (Jiang et al., 2018) but this remains to be confirmed.

Similar auxin-mediated internode elongation was shown in the maize mutant *brevis plant1* (*bv1*), where another type of inositol polyphosphate 5-phosphatase, also involved in the control of auxin signalling network, resulted in a semi-dwarf phenotype with very short internodes in mutant plants (Avila et al., 2016). In contrast, in another maize mutant, *d2014*, a novel gene, *BR2*, was identified, which encodes a P-glycoprotein-1 (PGP1) polypeptide from the group of AUX, auxin efflux proteins. Therefore, low auxin levels mediated by the BR2 protein can regulate internode elongation (Zhang et al., 2019).

Additionally, in rice mutants of *OsPP2C34*, a short internode phenotype was reversed in response to GA and upon overexpression of *GA2ox1* (Hossain et al., 2018). In melon (*Cucumis melo* L.), the newly identified gene *CmSi* (short internode) from the ERECTA gene family was reported as a positive regulator of internode and stem elongation under auxin regulation (Yang et al., 2020).

### Suppressor of Overexpression of Constants 1 (*SOC1*), MADS box transcription factor gene

In *Medicago truncatula*, a *Mtsoc1a* mutant was reported to be showing delayed flowering and short primary stems with shorter nodes to the first flower (Jaudal et al., 2018; Table 3). Overexpression of *MtSOC1a* caused a dramatic increase in primary stem height, promoting internode cell elongation in the primary stem. The *MtSOC1a* (Medtr7g075870) gene represents the transcription factor gene *Suppressor of Overexpression of Constants 1 (SOC1)*, belonging to the MADS-box family 22 and entitled MIKC with four abbreviated domains (MADS, intervening, keratin-like and C-terminal). The *SOC1* gene is a member of a complex regulatory network for flower and reproductive development. However, in the *Mtsoc1a* mutant, the homolog of the gene resulted not only in delay of flowering time but also in internode elongation (Jaudal et al., 2018).

Similar results were observed in other plant species, i.e., in rice where knockout of *OsMADS50*, the orthologue of *Arabidopsis SOC1* genes, showed an increased number of nodes and elongated internodes and therefore taller plants (Lee et al., 2004). In contrast, in maize, *ZmSOC1* overexpression lines (back-crossed with WT) were significantly shorter in height and had fewer stem nodes and leaves (Song et al., 2021). Similar results were reported in transgenic rice overexpressing *BoMADS50* (*BoSOC1*) from the woody bamboo species, *Bambusa oldhamii* (Hou et al., 2021).

### *BEBT* gene (benzyl alcohol *O*-benzoyltransferase)

The candidate gene *BEBT* (benzyl alcohol *O*-benzoyltransferase) was identified for the HFP trait in a Brazilian diversity panel of 178 accessions of common bean using a Genotyping-by-sequencing (GBS) approach (Delfini et al., 2021) (Table 3). Four Quantitative trait nucleotides (QTNs) in total were detected as involved in HFP, but only one QTN, with the *BEBT* candidate gene, showed the highest LOD score (3.84–6.86) (Delfini et al., 2021).

The enzyme benzyl alcohol *O*-benzoyltransferase (2.3.1.196), encoded by the candidate gene *BEBT*, is a member of the BAHD superfamily of CoA-dependent acyltransferases (D’Auria, 2006). The major role of BAHD enzymes is the synthesis of cell wall components, cutin, suberin and waxes (Molina and Kosma, 2015), fat biosynthesis in cacao seeds (Abdullah et al., 2021), and flavonoid acylation, including anthocyanin (Bontpart et al., 2015). *BEBT* has a very different function in the production of volatile esters and aroma formation during fruit development in pear and other horticultural species (Liu et al., 2020), and in flowers of the fairy fans [*Clarkia breweri* (Gray) Greene] (D’Auria et al., 2002).

The genes in the BAHD superfamily are conserved among most plant species (Yu et al., 2009) but are absent in the model plant species *Arabidopsis* (D’Auria et al., 2002; D’Auria, 2006). Nevertheless, there is no indication for morphological changes in cell growth, their number or elongation. The very diverse functions of *BEBT* genes make it difficult to determine which biochemical pathway could be related to the strong effect of the QTN identified in the common bean GWAS study (Delfini et al., 2021), and further molecular and biochemical studies are required.

### *WD40* gene (WD40-repeat protein)

The QTN analysis based on GWAS showed another candidate gene closest to the identified SNP. This gene, *Phvul.006G098200*, was annotated as a WD40-repeat gene (*WD40* or *WDR*) with unknown function, and we considered it as an alternative candidate gene for the HFP trait (Table 3).

The analysis of the publicly available physical map of the common bean on the Phytozome website^1^ and the LIS website^2^ indicated by MacQueen et al. (2020) revealed that the candidate gene *BEBT* (*Phvul.006G098300*) showed very low expression in all tissues of common bean accessions. In addition, none of the seven tandemly repeated copies of *BEBT* genes, proximal to the identified *Phvul.006G098300* (*Phvul.006G098400 - Phvul.006G099900*), were expressed in any tissue.

Delfini et al. (2021) indicated in their article about the *BEBT* candidate gene in common bean that the linkage disequilibrium (LD) block around the significant SNP was very large and determined the average 296K bp to select potential candidate genes in specific QTN distance. However, in our search, the alternative candidate gene, *Phvul.006G098200*, was located within 1% of the genetic distance in the distal direction about 3K bp from the *BEBT*.

This gene was annotated as a WD40-repeat gene (*WD40* or *WDR*), and our further analysis indicated for different type of the expression. In contrast to the previous candidate, the *WD40* gene was moderately expressed in all tissues including roots, nodules, leaves, stems, and pods, but not in flower buds and flowers^3^.

WD40 proteins contain a typical Trp-Asp motif with highly conserved repeating units (Neer et al., 1994), and they play a regulatory role in a diverse range of functions in plants including meristem and floral development, light signalling, cell division, cytokinesis, and apoptosis (van Nocker and Ludwig, 2003). A phosphatase protein with several WD40 repeats interacts with protein kinase (SnRK1) and regulates *Arabidopsis* plant growth in conditions of different nutrient availabilities (Ananieva et al., 2008), but it was not studied in legumes.

In rice (*Oryza sativa* L.), 200 *OsWD40* genes were identified, which fall into five distinct molecular-phylogenetic clades and 11 subfamilies (Ouyang et al., 2012). After BLAST-P comparison of the WD40 polypeptide (Phvul.006G098200) from common bean with the rice annotated protein with WD and G-beta repeat domains *via* the rice genome web-site^4^, LOC_Os12g06810 was identified as the closest match with 81% similarity and 69% identity. The corresponding gene was named *OsWD40-194*, and Affymetrix microarray analyses revealed moderate expression profiles in all tissues except developing seeds and seedlings (Ouyang et al., 2012).

However, more attention must be paid to another rice gene, *OsLIS-L1* (Lissencephaly type-1-like), that encodes a protein with a WD40-repeat domain (LOC_Os08g06480) (Gao et al., 2012). This gene named *OsWD40-155* was assigned to a different clade of the molecular dendrogram and was highly expressed during panicle development (Ouyang et al., 2012). Most importantly, mutants of the gene *OsLIS-L1* = *OsWD40-155*, had the length of the first internode under the panicle shorter (22 cm) compared to the WT (32 cm). Importantly, no significant differences were found in the lengths of other corresponding internodes (Gao et al., 2012).

The gene *OsLIS-L1 = OsWD40-155*, however, reduced the length of the highest internode in the stem under the panicle of rice plants and that the length of the lowest internodes, of particular interest to this review, remained unchanged. Nevertheless, despite the effects on different parts of the stem in rice plants, *WD40* potentially can be recognised as a candidate gene for HFP in legume plant species, subject to further evaluation and functional validation.

## Summarised model of height to first pod in legumes

**FIGURE 4:**
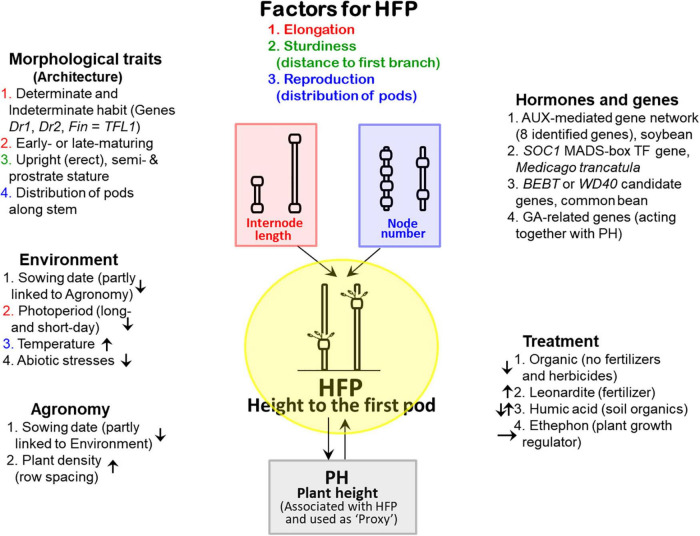
Schematic representation summarising the model of HFP, and how and which other traits, factors, and genes interact and influence HFP. At the top, three major factors are indicated corresponding to internode length (red) and node number (blue), which form the basis of the mechanism of HFP formation; the factor in green represents a combination of both (red and blue). At the bottom, PH is indicated as the associated ‘proxy’ with HFP. Three sections on the left-hand side show the roles of morphological traits, environmental factors, and agronomic management in HFP formation and performance. On the right-hand side, two other sections represent hormones and candidate genes identified to be controlling the development of HFP in legume species with treatment of some substances shown to have effects on HFP. Coloured numbers of the sections correspond to major factors. Arrows presented inside of sections indicate the effect (stronger or longer) of the component on the increase in HFP (‘↑‘), decrease in HFP (‘↓’), or no effect on HFP (‘→’).

The summary model is presented in Figure 4. As shown in the review, HFP is based on two traits, internode length and node number (Figure 1). The interaction of all the other traits and candidate genes can be mediated *via* three major factors as described earlier: (1) elongation factor for internodes, (2) sturdiness factor for length from soil to the first branch, and (3) reproductive factor with nodal distribution of pods on the stem (Acquaah et al., 1992; Kelly, 2001). This is the fundamental mechanism of HFP formation and for the analysis of other traits influencing HFP. Obviously, the first factor (elongation) and the third one (reproduction) are related to internode elongation and node number, respectively. The second factor (sturdiness) represents both factors 1 and 3. At the same time, PH is a very important trait for HFP, and the traits interact in both ways. This is because HSP is a part of PH while PH can be a ‘proxy’ that is associated with HFP.

Four main morphological traits are described in the review, and they are all related to plant architecture, control of growth type and plant stature, distribution of pods on the stem, and maturation. All of these morphological traits have a clear influence on the three major factors mentioned above. Of the environmental factors, two of them (photoperiod and temperature) are shown to be involved in internode elongation and formation of nodes with pods, respectively. The sowing date, in fact, is a shared role of agricultural practice, and later sowing dates cause shorter HFP in the studied legume plants. In general, abiotic stress affects entire plant growth, and PH and HFP as well. However, this is a rather unspecific impact, with abiotic stress affecting all traits including HFP. In agronomy, plant density and row spacing showed a very strong influence with increased HFP and was associated with higher density of plants in field trials.

Hormones and candidate genes were identified as controlling the development of HFP in legume species. However, this piece of research is not completed yet, and all conclusions are only preliminary. Nevertheless, each of the proposed candidate genes, singularly and collectively, may be extremely important keys for a clearer understanding of the molecular and genetic mechanisms of HFP formation and development in future studies. Finally, treatments with various substances such as fertilisers, soil organic matter, and plant growth regulators have various effects on HFP, where the exact mechanism of the used substances on HFP remains unclear. Results with organic agriculture was also included here just to show the effect on lack of fertilisers and herbicides.

The view of HFP summarised in Figure 4 is still fragmented, and not all ‘pieces of the puzzle’ are identified and described properly. However, this is the first attempt to combine all known information together about HFP in legume crops, which can greatly assist in the study of this subject in the future.

## Height to first pod and legume breeding

As indicated in this review, legume cultivars with low HFP can result in significant losses in seed yield during mechanical combine harvesting. Therefore, aiming for higher HFP would be favourable as a breeding strategy. However, while the long-term breeding strategy for soybean cultivars released during the last century in various regions of China would be expected to show a gradual increase in HFP where genotypes with higher position of the first pod may have been preferred by breeders, in fact, no significant differences in HFP were reported (Wu et al., 2015). More variable results for HFP among Chinese soybean cultivars released over 60 years were presented in another report. HFP was decreased in the north spring growing region, increased in the Yellow-Huai-Hai summer growing region, and remained unchanged in the southern region of China (Qin et al., 2017). Yield was not consistently related to HFP, indicating that other traits can have a more important role in seed production in soybean depending on climate and environment (Qin et al., 2017).

Additionally, changes in harvesting technology, from primitive tools to modern harvesters, might also have contributed differently to an HFP shift during long-term breeding. In this context, there has been tremendous progress in modern harvesting combines and machinery in the last few years, i.e., ‘flex head’ combines, which can harvest plants leaving very low remaining stubble (example review in rice: Fu et al., 2022). However, this subject is beyond the aim of the current review.

Nevertheless, it is also important to better understand the biological mechanisms behind seed formation in the first-developed (earlier) and next (later) sets of flowers. The published results in lentil suggested that seed yield was higher from earlier set of flowers, where the set was 2.6 flowers per node. Later-developed flowers at higher nodes on the stem of lentil plants had 2.1 flowers per node and smaller yields were recorded (Bueckert et al., 2020). Similar results were reported for vegetable pea, where genotypes producing flowers and pods earlier on lower nodes tended to yield better than those with later flowers and pods at higher nodes (Singh and Dhall, 2018). Therefore, HFP must be balanced with other traits for plant architecture and growth, distribution of pods along the stem, and PH, which is more or less positively correlated with HFP, and can also contribute to lodging resistance if more pods are grown higher on the stem (Wu et al., 2015).

The studies presented here show genetic diversity for HFP among reported accessions of legume plants. The heritability is also diverse and depends upon many other genetic and environmental factors and connected with PH. How could changes in HFP be associated with improvement in seed yield in legume crops?

In plants with generally higher HFP, low or no further improvement in seed yield was reported. For example, higher or lower HFP in six soybean cultivars did not show differences in seed yield in any field plots regardless of conventional or organic cropping systems in Germany (Beiküfner et al., 2019). In chickpea, increased HFP had a positive but not strong effect on seed yield per plot (Petrova, 2021). Low correlations (*r* = 0.20–0.118) between HFP and seed yield per plot or per plant was reported in different experiments among 9 and 7 common bean cultivars (Eckert et al., 2011a; Girgel, 2021), and even negative correlations (*r* = −0.275 and −0.265) were found in 12 pea genotypes and in 4,050 mutants of chickpea plants, respectively (Togay et al., 2008; Amri-Tiliouine et al., 2018). A similar negative correlation (*r* = −0.15) was reported between HFP and seed yield in 570 soybean germplasm collections from 24 countries evaluated during 27 years in the Far-East region of Russia (Seferova and Bulakh, 2019). In faba bean, a negative correlation was also found between HFP and three indices of seed production per plant, ranging from *r* = −0.137 to -0.404 (Kosev and Georgieva, 2021). In contrast, in chickpea, plants with HFP of 25–29 cm had a positive impact on seed yield per square metre and showed an especially strong improvement (by 36.5%) in seed yield in Bulgarian accessions (Petrova, 2021). Therefore, the relationship between HFP and seed yield from mechanical harvesting may be variable between species and under different field planting conditions.

Gene pool enrichment of modern cultivars and breeding lines may be achieved by introgressing wild species by hybridisation and selection of potential favourable recombinants, with many excellent practical results of improved breeding for seed yield in legumes (reviewed in Pratap et al., 2021), and the potential for its application to improve HFP. However, this strategy may not be always successful. For example, despite very high genetic variability, the wild annual species *Cicer* did not provide any advantage for genetic improvement of HFP and seed production in chickpea from recombinant analyses of interspecific crosses (Robertson et al., 1997). In contrast, in wild landraces of common beans in Turkey, HFP showed a highly significant and positive correlation with seed yield per plant, suggesting that this trait possibly can be utilised for breeding of superior common bean genotypes (Nadeem et al., 2020). Nevertheless, potential success may be very limited in common bean because most wild landraces have a climbing growth habit, while the preferred upright plant architecture is only present in very limited specific genetic groups and market classes (Checa and Blair, 2008; Aragão et al., 2011).

Traditional breeding methods were successfully used in the production of the modern Korean soybean cv. Saegeum with 18 cm HFP using the single seed descent method in F_3_-F_5_, and the best breeding lines were selected by pedigree in F_6_-F_7_ progenies. Saegeum was well-adapted to mechanised harvesting, with high HFP compared to the local standard Daewonkong with 11 cm HFP (Kim et al., 2019). However, this is a rare case in the recent literature describing new cultivar production using classical breeding methods with significantly increased HFP.

A more striking example was shown in the breeding strategy of common bean, starting from gamma-radiation produced mutants more than 60 years ago (Kelly, 2001) with subsequent long-term recurrent selection which resulted in the development of new upright varieties. Currently, the vast majority of modern varieties of common bean have an erect plant architecture and upright canopy, which is very suitable for direct harvest. Therefore, it also enables simpler harvesting in a single pass with minimal seed loss during direct harvest compared to the older conventional harvesting style requiring two passes (Eckert et al., 2011a; Soltani et al., 2016). In this regard, the higher distribution of pods in the middle and upper parts of stem in modern upright varieties of common bean illustrates the great success that can result from genetic improvement of plant stature and development traits, of which HFP and pod distribution along the stem are key factors.

Modern genetic and molecular methods are being developed very actively for legume crop breeding, where next-generation sequencing is widely applied and genomes of some species are fully sequenced. Based on that, development of various molecular markers and marker-assisted selection (MAS) represent a relatively simple technology as the initial steps of more advanced modern genomic selection in many legume plant species (reviewed in Varshney et al., 2013).

However, in molecular research, little attention has been paid to HFP, a trait that does not fit neatly into the categories of disease resistance or tolerance to abiotic stress. The HFP trait is only related to combine harvesting; therefore, its study has lagged far behind that of other traits in legumes at the level of genomic selection. Nevertheless, some molecular methods can also be successfully used to increase HFP in legume plant species. The results presented in this review show some QTLs based on mutants, hybridisation, and GWAS analyses (Table 3). However, a more comprehensive study is required from QTLs to candidate gene identification. For example, a QTL for stem internode length was mapped to chromosome 9 in Adzuki bean [*Vigna angularis* (Willd.) Ohwi and H. Ohashi] (Li et al., 2017) and two QTLs to chromosomes 1 and 5 in lentil (Fratini et al., 2007), while two QTLs for node number to first flower were mapped to chromosomes LG11 and LG4 in asparagus bean [*Vigna unguiculata* ssp. *sesquipedalis* (L.) Walp] (Xu et al., 2013). However, further analyses are required for QTLs and meta-QTLs, and synteny studies on legumes to identify and realise candidate genes in practical breeding of legumes.

In the current review, QTL analysis, mapping, and identification of possible candidate genes are reported for HFP (Table 3). They included 11 major QTLs with eight candidate genes for HFP involved in auxin transport and signal transduction in soybean (Jiang et al., 2018): the MADS box gene *SOC1* in *Medicago trancatula* (Jaudal et al., 2018) and *BEBT* or *WD40* genes located nearby in the mapped QTL in common bean (Delfini et al., 2021). All or some of these are likely to control HFP with either clear or yet to be defined mechanisms, and future research should help to understand better the molecular-genetic control of this trait.

Of course, the identification and expression analysis of candidate genes are very important steps, and modern ‘omics’ technology can be especially helpful in legumes, including proteomics (reviewed in Jan et al., 2022). However, for practical breeding in legumes for increased HFP, simple and effective molecular markers and corresponding MAS are the most direct path to the identification of promising breeding lines. None of the reports on genes involved in controlling HFP contain information about development, analysis, and validation of simple diagnostic markers associated with the trait. The analysis is necessary if any of the genes could be used for marker development and MAS for this trait in practical breeding.

Several reviews (Kumar et al., 2018; Dhaliwal et al., 2020) and books (Gupta et al., 2014; Gosal and Wani, 2020) were published about molecular marker-assisted gene pyramiding in legumes containing very little information about any markers including RAPD, ISSR, AFLP, SSR, and SNP markers surrounding the mapped HFP QTLs (Fratini et al., 2007; Xu et al., 2013). A very initial study on SNP markers in common bean revealed the presence of a considerable genetic variation among the assessed genotypes (Nkhata et al., 2020), which is probably important for future research but not so useful for MAS in practical breeding. Therefore, in the current situation, the proposition for a breeding strategy for HFP in legume crops cannot be based on molecular markers since they are not yet developed but only for the combination and interaction of other traits, as summarised in Figure 4. In contrast to legumes, in kenaf (*Hibiscus cannabinus* L.), the SLAF marker M41961 with an identified SNP was successfully developed for the first flower node trait with an accuracy rate of 91% (Li et al., 2019). Therefore, further research in this area in legumes is warranted and could realise significant yield increases that will be needed in the future.

## Author contributions

MK and GK: writing parts of the introduction and terminology. IO and ET: internode length and node number. SJ: phenotypic variability and heritability. RY, KB, and SK: breeding strategy. CSc: external factors. PA: type of stem growth. CSw and YS: genes and QTLs. CJ: helped with the illustrations. KS: supervised the project. YS: coordinated the study and prepared the initial draft of the manuscript. CSc, PA, CSw, CJ, and KS: edited the manuscript. All authors reviewed and approved the final version of the manuscript.

## Footnotes

1
https://phytozome-next.jgi.doe.gov/report/transcript/Pvulgaris5_593_v1_1/Pv5-593.06G105300.1

2
https://legacy.legumeinfo.org/feature/Phaseolus/vulgaris/gene/phavu.G19833.gnm1.ann1.Phvul.006G098300

3
https://phytozome-next.jgi.doe.gov/report/transcript/Pvulgaris5_593_v1_1/Pv5-593.06G105200.3

4
http://rice.uga.edu
