# Supplementary material for: Height to first pod: A review of genetic and breeding approaches to improve combine harvesting in legume crops
Source: Front Plant Sci. 2022 Sep 16;13:948099. doi: 10.3389/fpls.2022.948099 (PMC9523450; doi:10.3389/fpls.2022.948099)
Supplement: Supplementary file 1 [file Table_1.pdf]

Supplementary Material

Table S1. Variability and heritability (H^2 or h^2) of height to first pod (HFP) and correlation (*r*) with plant height (PH) in legume plant species.

HFP (cm)	Recommended HFP (cm)	H^2 or h^2 (Broad and narrow sense)	*r* (HFP/ PH)	Description	Reference
**Soybean [*Glycine max* (L.) Merr]**					
14.4-32.0	15	H^2 = 0.50-0.74 (F_2) and 0.29-0.63 (F_3)	*r*=0.25 -0.30	Three crosses between cultivars with different habit of stem growth. Progenies F_2 and F_3 were studied in field trials in the USA.	Martin and Wilcox, 1973
12.7-16.5; 24.9-31.0				1 cultivar grown during 2 years in one location in Turkey.	Yilmaz et al., 2003
		H^2 = 0.63 and 0.31 (hybrids & families)		F_2 genotypes and corresponding F_3 offspring from six populations in field trials in Brazil.	Costa et al. 2008
6.4-10.0; 10.0-12.5				2 cultivars grown during two years in three locations in the USA.	Epler and Staggenborg, 2008
10.0-14.1 11.4-14.1				1 cultivar with different density and fertiliser grown during 2 years in one location in Turkey.	Öz, 2008
7.0-24.2	12		*r*=0.47	90 cultivars, two consequent vegetation periods in India.	Ramteke et al., 2012
8.1-21.1	15	H^2 = 0.51-0.79	*r*=0.61	4 cultivars and three hybrid populations grown in Korea.	Kang et al., 2017
20.8-32.5		H^2 = 0.00		2 parents and F_2 hybrids from Brazil were grown in greenhouse.	Teixeira et al., 2017
17.0-20.1; 14.6-23.4				3 genotypes with determinate and one genotype with indeterminate growth from Japan.	Kato et al., 2018
5.6-9.4; 7.9-12.8			*r*=0.67	6 cultivars grown in organic and conventional cropping systems during three years in Germany.	Beiküfner et al., 2019
13.7-17.9				Various genotypes with determinate, semi-determinate and indeterminate growth from Japan.	Kato et al., 2019
6.0-22.0			*r*=0.23	570 germplasm collection from 24 countries, evaluation during 27 years.	Seferova and Bulakh, 2019
9.4-13.4; 10.3-13.4	>10			4 cultivars with different sowing density or seeding systems, grown in two years and two locations in Germany.	Sobko et al., 2019, 2020

11.9-35.7				12 cultivars from Turkey.	Celik and Boydak, 2020
	15			1 cultivar with different sowing density grown during three years in Poland.	Rębilas et al., 2020
7.0-11.7; 5.8-12.8			r=0.71	4 cultivars from Europe, grown in different years.	Borowska and Prusiński, 2021
14.1-35.9		H^2 = 0.33		16 germplasm accessions from Nepal.	Khadka et al., 2021
7.7-9.5				6 cultivars without and with inoculation from Serbia.	Miladinović et al., 2021
14-23				15 cultivars with three maturity groups from Poland.	Staniak et al., 2021
12.1-17.2				10 cultivars with various maturity and groups and growth habit during two years from Brazil.	Santana et al., 2022
**Common bean (*Phaseolus vulgaris* L.)**					
1.34-2.29				3 pinto cultivars from the USA grown in four environment during two years in the USA with three row specings.	Eckert et al., 2011b
14.0-35.5; 11.6-32.7				26 genotypes from Brazil grown in two locations.	Zilio et al., 2013
14.0-21.5	15		r=0.57	1 cv. IPR Tuiuiú, in humid subtropical environment of Brazil.	Bisognin et al., 2019
6.4-68.9		h^2 = 0.92	r=0.69	180 wild accessions and 3 cultivars grown in two locations and 5 environments from Turkey.	Nadeem et al., 2020
15.2-19.6		h^2 = 0.68	r=0.52	178 accessions representing Brazilian diversity panel, in four environments during two years.	Delfini et al., 2021
15.4-18.7			r=0.43	7 cultivars from Turkey.	Girgel, 2021
**Chickpea (*Cicer arietinum* L.)**					
1.0-11.5; 10.0-22.5				228 accessions of eight annual wild *Cicer* species and 20 cultivated Kabuli chickpea lines in ICARDA, Syria.	Robertson et al., 1997
19.9-21.9; 22.0-27.1				7 cultivars (1 cultivar ecotype Desi and 6 cultivars ecotype Kabuli), with various plant density, tested in two geographic locations during 2-3 years in Canada.	Gan et al., 2003a
30.1-32.6				I cultivar (Kabuli ecotype) in experiment with seed size and seeding depth during three years in Canada.	Gan et al., 2003b
		H^2 = 0.31		15 Kabuli ecotype accessions during two tests in Turkey.	Özveren et al., 2006
		H^2 = 0.83; h^2 = 0.67		4 cultivars and reciprocal F_1 hybrids.	Biçer and Şakar, 2008

		H^2 = 0.70; h^2 = 0.47		Half-diallel crosses between five Kabuli accessions in Iran.	Karami, 2011
13.7-19.1; 14.4-18.3				Two and four cultivars treated with humic acid during two years in Turkey.	Ulukan et al., 2012a, 2012b
17.2-29.1			*r*=0.24	4,050 mutant plants (135 M_2 families).	Amri-Tiliouine et al., 2018
19-38	25-29			95 accessions with various origins.	Petrova, 2021
**Pea (*Pisum sativum* L.)**					
10.8-71.5				9 genotypes from India.	Gupta et al., 1983
			r=0.83	12 genotypes in Turkey	Togay et al., 2008
42.2-79.8		h^2 = 0.68-0.70		2 parents and segregating populations with morphologically contrasting characteristics for growth habits in Colombia.	Gómez and Ligarreto, 2012
			r=0.89	9 spring-sown Bulgarian and Ukrainian field pea cultivars.	Kosev and Mikić, 2012
12.5-42.9		H^2 = 0.93		55 genotypes produced and grown in India.	Singh et al., 2019
20.8-56.1				10 pea genotypes from Bulgaria.	Kalapchieva et al., 2020
**Faba bean (*Vicea faba* L.)**					
6-41				13 local genotypes from Albania.	Nasto et al., 2016
		H^2 = 0.97	*r*=0.84 -0.86	100 indigenous and exotic genotypes grown during two years in India.	Bora et al., 1998
22-35				17 accessions grown during two years in Bulgaria.	Kosev and Georgieva, 2021
6.0-13.6				Plants cv. Salkim, used for treatment by organic fertilizer leonardite.	Uçar et al., 2021
**Lentil (*Lens culinaris* Medik.)**					
7.6-11.7		h^2 = 0.28	*r*=0.60	140 M_2 families with cv. Idlib-3 background in Algeria.	Tabti et al., 2018
5.8-13.3		H^2 = 0.55		11 germplasm accessions from ICARDA grown in 3 locations in Iraq.	Ahmad et al., 2021
**Cowpea [*Vigna unguiculata* (L.) Walp.]**					
36.6-63.2				2 cultivars and 7 breeding lines grown during two years in two location in Turkey	Basaran et al., 2011
10.0-45.0				1 cultivar with different sowing density grown during three years in Brazil	Sorrato et al., 2020

**Bitter vetch [*Vicia ervilia* (L.) Willd.]**					
5.1-11.8				42 landraces from Iran and Azerbaijan.	Hassanpour and Sahhafi, 2020
**Cluster bean, guar [*Cyamopsis tetragonoloba* (L.) Taub.]**					
2.5-6.2			*r*=0.71	8 accessions of from South Africa, India and the US grown in Italy.	Gresta et al., 2016
**Fenugreek (*Trigonella foenum-graceum* L.)**					
25.3-41.8				10 accessions grown during two years in Turkey.	Güzel and Özyazıcı, 2021
